# Cross-diagnostic validity in a generic instrument: an example from the Functional Independence Measure in Scandinavia

**DOI:** 10.1186/1477-7525-4-55

**Published:** 2006-08-23

**Authors:** Å Lundgren-Nilsson, A Tennant, G Grimby, KS Sunnerhagen

**Affiliations:** 1 Department of Rehabilitation Medicine, Academic Unit of Musculoskeletal Disease, The University of Leeds, 36 Clarendon Road, Leeds, LS2 9NZ, UK; 2 Sahlgrenska Academy at Göteborg University, Institute of Neuroscience and Physiology/Rehabilitation medicine, Guldhedsgatan 19 413 45 Göteborg, Sweden

## Abstract

**Background:**

To analyse the cross-diagnostic validity of the Functional Independence Measure (FIM™) motor items in patients with spinal cord injury, stroke and traumatic brain injury and the comparability of summed scores between these diagnoses.

**Methods:**

Data from 471 patients on FIM™ motor items at admission (stroke 157, spinal cord injury 157 and traumatic brain injury 157), age range 11–90 years and 70 % male in nine rehabilitation facilities in Scandinavia, were fitted to the Rasch model. A detailed analysis of scoring functions of the seven categories of the FIM™ motor items was made prior to testing fit to the model. Categories were re-scored where necessary. Fit to the model was assessed initially within diagnosis and then in the pooled data. Analysis of Differential Item Functioning (DIF) was undertaken in the pooled data for the FIM™ motor scale. Comparability of sum scores between diagnoses was tested by Test Equating.

**Results:**

The present seven category scoring system for the FIM™ motor items was found to be invalid, necessitating extensive rescoring. Despite rescoring, the item-trait interaction fit statistic was significant and two individual items showed misfit to the model, Eating and Bladder management. DIF was also found for Spinal Cord Injury, compared with the other two diagnoses. After adjustment, it was possible to make appropriate comparisons of sum scores between the three diagnoses.

**Conclusion:**

The seven-category response function is a problem for the FIM™ instrument, and a reduction of responses might increase the validity of the instrument. Likewise, the removal of items that do not fit the underlying trait would improve the validity of the scale in these groups. Cross-diagnostic DIF is also a problem but for clinical use sum scores on group data in a generic instrument such as the FIM™ can be compared with appropriate adjustments. Thus, when planning interventions (group or individual), developing rehabilitation programs or comparing patient achievements in individual items, cross-diagnostic DIF must be taken into account.

## Background

Medical outcome studies use generic instruments to compare results between different settings with different case mixes. It is generally thought that they give less information about each patient group, but it has also been suggested that well designed generic instruments may be at least as good as some disease specific instruments [1]. Although many such measures are available, their use in clinical practice in Europe is limited [2]. While the demands of clinical management in a hospital setting requires measures of outcome, there are several factors that may influence which measure is chosen. For example, within Europe, outcome measures will need to be adapted to a particular language [3], and there may thus be a preference for outcome measures that already have a local adaptation. The emergence of new techniques to evaluate the invariance of instrumentation across groups has provided the opportunity to compare measures used within and across diagnostic groups at both national and international levels in rehabilitation [4]. The FIM™ is mainly a measure of activity limitation that is used across a wide range of conditions and in a variety of situations in rehabilitation. Assessments are usually made through observations and the scores are set by consent by the team members. FIM™ can also be used individually by any member of the team. It was designed to measure level of disability regardless of the nature or extent of the underlying pathology or impairment [5] where a change in the sum score reflects the gain in independence. The Uniform Data System (UDS) is a central databank facility in Buffalo to which individual rehabilitation units submit their data for comparative purposes. The implementation of such an approach has limitations in that it requires a substantial (and continuing) investment in quality control, training and access to a central facility. The validity and reliability of the FIM™ have been described in reports using different methods [6,7]. Comparisons across countries in Europe within diagnostic groups have already been made [4,8,9]. In the present study we consider the health care system, social environment, hospital settings and culture to be similar enough that it is acceptable to pool data in Scandinavia.

The Scandinavian countries have a common socio-cultural background. The health care system is very similar with taxed financed service. Health professionals work across borders and also patients are treated over the borders. Thereby we argue that the differences are smaller than between states in the USA.

This paper is concerned with the cross-diagnostic validity of the motor items of FIM™ in three neurological diagnoses, Stroke, Traumatic Brain Injury (TBI) and Spinal Cord Injury (SCI).

## Methods

Admission data from the nine participating Scandinavian rehabilitation units (one Norwegian, one Danish, seven Swedish), members of the Pro-ESOR [2] study on in-patients, were used. From this an equal sample (n = 157) from each diagnosis was used taken from a total sample of 1661 (stroke 736, SCI 358, TBI 567). For patients with stroke data came from Sweden and Norway. The Spinal Cord Injury (SCI) data came from Denmark and data on patients with TBI from Sweden.

### Functional Independence Measure

The FIM™ consists of 13 motor and 5 social-cognitive items, assessing self-care, sphincter, management, transfer, locomotion, communication, social interaction and cognition [5,10]. It uses a 7-level scale anchored by extreme rating of total dependence as 1 and complete independence as 7; the intermediate levels are: 6 modified independence, 5 supervision or set up, 4 minimal contact assistance or the subject expends >75% of the effort, 3 moderate assistance or the subjects expends 50 to 74% of the effort, and 2 maximal assistance or the subject expends 25 to 49% of the effort.

The FIM™ was originally developed as an 18-item scale, but it was later shown that it was possible to treat it as two separate scales, a 13-item motor and a 5-item social-cognitive scale [11]. The present study used only data from the FIM™ motor scale. Data were collected on admission according to the FIM™ manual. FIM™ has been used in Sweden since 1991 and training has been given to new users. Training was also given to the Norwegian centres and Denmark. The centres did however not have to state which version of the manual was used, however the manuals are quite similar.

### Rasch analysis

The Rasch model [12] was used as the methodological basis for examining the internal construct validity, the scaling properties of the FIM™ motor items, the possibility of a sum comparison between diagnoses and, where appropriate, through analysis of Differential Item Functioning (DIF), its cross-diagnostic validity. The Rasch model is a unidimensional model that asserts that the easier the item, the more likely it will be affirmed, and the more able the person, the more likely he or she will affirm an item compared with a less able person. The model used in the present study is the Partial Credit Model [13] chosen after testing if the data met the assumption of the Rating Scale Model with Fisher's likelihood ratio test between the two models :

ln⁡(Pnik1−Pnik−1)=θn−bik
 MathType@MTEF@5@5@+=feaafiart1ev1aaatCvAUfKttLearuWrP9MDH5MBPbIqV92AaeXatLxBI9gBaebbnrfifHhDYfgasaacH8akY=wiFfYdH8Gipec8Eeeu0xXdbba9frFj0=OqFfea0dXdd9vqai=hGuQ8kuc9pgc9s8qqaq=dirpe0xb9q8qiLsFr0=vr0=vr0dc8meaabaqaciaacaGaaeqabaqabeGadaaakeaacyGGSbaBcqGGUbGBdaqadiqaamaalaaabaGaemiuaa1aaSbaaSqaaiabd6gaUjabdMgaPjabdUgaRbqabaaakeaacqaIXaqmcqGHsislcqWGqbaudaWgaaWcbaGaemOBa4MaemyAaKMaem4AaSMaeyOeI0IaeGymaedabeaaaaaakiaawIcacaGLPaaacqGH9aqpiiGacqWF4oqCdaWgaaWcbaGaemOBa4gabeaakiabgkHiTiabdkgaInaaBaaaleaacqWGPbqAcqWGRbWAaeqaaaaa@4941@

which is the log-odds of person *n *affirming category *k *in item *i*; *θ *is person ability, *b *is the item difficulty parameter, *τ*_*k *_is the difficulty of the *k *threshold, and *P*_*nik *_is the probability for person *n *to answer item *i *in category *k*. The units of measurement obtained form the equation are called "logits", which is a contraction of log-odds probability units. When the observed response pattern coincides with or does not deviate too much from the expected response pattern, then the items constitute a true Rasch scale [14]. Test of fit to the Rasch model is preceded by a number of overall tests and by tests of fit for individual items. The latter are given in the form of residual values (the standardised difference between the observed and the expected score for each person), which should be between -2.5 and 2.5 [15], and Chi-Square statistics, which should show non-significant deviation from the model expectation. The Chi-Square values are calculated on the basis of ability groups (or Class Intervals) of approximately 50 people to which the patients are assigned on the basis of their total score. Three overall summary fit statistics are given; 1) Overall item and 2) person fit statistics approximate a normal distribution with a mean of 0 and standard deviation of 1 when data fit the model and 3) An item trait interaction statistic which tests that the hierarchical ordering of the items remains the same for discrete groups of patients across the trait. This is reported as a chi-square statistic, and probability should be greater than 0.05 (no significant difference).

Due to the number of tests of fit undertaken (e.g. 13 for each item in the motor scale) Bonferroni corrections were applied giving a significant p-value of 0.004 for the motor FIM™ [16]. In addition to these overall fit statistics a Person Separation Index (PSI) is calculated as the base for estimating internal consistency reliability, where the estimates on the logit scale for each person are used to calculate reliability. The interpretation is similar to Cronbach's ά. The PSI and indicates the degree to which the scale can separate patients into discrete groups. A value of 0.7 is the minimum required to discern two groups [17]. Finally, confirmation of local independence of items (no residual associations in the data after the Rasch trait has been removed) confirms unidimensionality [18].

### Analytical strategy and procedure

The first step in analysing the psychometric quality of the FIM™ motor items in the present study was to examine the use of the rating scale in each diagnosis, together with the hierarchical ordering of the items. Where disordered thresholds were found, categories were collapsed. The threshold represents the equal probability point between any two adjacent categories within an item. The threshold is the level at which the likelihood of failure to agree with or endorse a given response category below the threshold equates to the likelihood of agreeing with or endorsing the category above the threshold. Estimates should be correctly ordered (i.e. increasing in value) if the categories are being assigned in the intended way.

Where thresholds are disordered categories are collapsed and in the current study collapsing was done by using headings of the categories in the FIM™ manual and clinical judgement, keeping the categories at the ends and collapsing the middle ones. This was followed by analyses of individual item fit to the model where only positive residuals, above 2.5, were considered, since negative residuals do not threaten the construct but simply do not provide more information for the analysis. Item-trait fit was also taken into account. The same procedure was repeated for the pooled data.

The next step was an examination for DIF, a requirement of measurement is invariance across groups. Items that do not yield the same item response function for two or more groups display DIF and violate the requirement of unidimensionality [19]. Consequently it is possible to examine whether or not a scale works in the same way by contrasting the response function for each item across groups. For tests of DIF, a sample size of 200 or less has been suggested as adequate [20]. DIF may manifest itself as a constant difference between countries/diagnosis across the trait (Uniform DIF – the main effect), or as a variable difference, where the response function of the two groups cross over (Non-uniform DIF – the interaction effect). Both the country/diagnosis/clinical factor and the interaction with the Class Interval (level of the trait) might be significant in some cases, as with any ANOVA's main and interaction effects. Tukey's post hoc tests determine where the statistically significant differences are to be found where there are more than two groups. This process has been described in more detail in another paper [4].

Where DIF identified the items were substituted for a series of diagnosis-specific items (e.g. Bathing becomes Bathing – SCI, Bathing – stroke, etc.). For each diagnosis, only the scores observed in its corresponding item are considered, while the other items are assigned structural missing values. Subsequent analysis is undertaken on this expanded data set (i.e. original plus split items).

Finally, when data are found to fit the Rasch model, as defined by acceptable fit statistics and the absence of DIF, a test of the assumption of local independence is undertaken to confirm the unidimensionality of the scale. This is based upon an examination of the patterning in the residuals and the magnitude of the fist residual component in a Principal Component Analysis of the residuals. This analytical strategy has been described in detail in earlier studies [4,8,21-23]

An analysis of the clinical meaning of the DIF problem was then conducted by testing whether the meaning of the summed score reflected the same amount of independence between the SCI, TBI and stroke pooled data. This was done by test equating, a procedure used to place item parameter estimates on the same scale when multiple test forms are given to examinees [24]. In RUMM2020 test equating can be explored graphically by comparing the raw-score to logit transformation graph for each test, and tables are produced for the raw score logit estimate values, which can be exported for further analysis.

To achieve test equating the data are stacked and racked [25], creating one item block for each of the three diagnoses linked by a block of the "original" items for all diagnoses together. Thus the original item set creates the link by having all cases in a vertical set (stacked) and the diagnosis specific items are then replicated horizontally (racked) with structural missing values for those cases not of that diagnosis. This will give items with missing values for the unique diagnostic items, e.g. Eating SCI will have missing values for stroke and TBI patients. This approach is sustainable since the Rasch model allows missing values [26-28]. This means in this study that the item blocks for each diagnosis can be considered as multiple tests or instruments. The test equating was done after adjustment of disordered thresholds, with the same scoring model for all item blocks (diagnoses). The relationship between the logit value for the summed score between the item blocks (diagnosis) was visually inspected and statistically analysed, where a difference of more than 0,65 logits at the margins and 0,30 in the middle [29] was considered clinical relevant.

The Rasch analysis was carried out with the RUMM2020 software [30].

## Results

### Scaling properties and fit within diagnoses

In the current analysis we used the Partial Credit Model as the data did not meet the assumption of the Rating Scale Model with a significant likelihood ratio test between the two models (p = <.0000001). Separate analyses for the three diagnoses showed disordered thresholds in a majority of the items. These were consequently rescored. All item categories were reduced to three in all diagnoses. This gave the new category 1 (old categories 1 and 2), new category 2 (old categories 3+4+5), and new category 3 (old categories 6 and 7). However this was not sufficient for some items. For SCI, two items had to be dichotomised, Grooming and Stairs, the latter was also dichotomised in TBI. For stroke, Bladder management and Bowel management had to be dichotomised. After rescoring, the items for stroke and TBI fitted the model. It was found that items Bladder management and Bowel management in SCI showed misfit to model expectations. Only the SCI data had a significant item-trait interaction. The person separation index was between 0.94 and 0.96 in the three diagnoses.

### Pooled data and cross-diagnostic DIF

Disordered thresholds were found in almost all items in the pooled data. After rescoring the majority of the items had three categories although Bladder management and Stairs had to be dichotomised. The items Eating and Bowel management showed individual misfit to the model. The summary item-trait interaction statistic also showed misfit. The person separation index was 0.95.

The data were then examined for cross-diagnostic DIF. All items showed DIF, and Tukey's post hoc comparison of these items revealed a complex pattern where 9 out of 13 items displayed DIF for SCI and 2 for TBI against the two other diagnoses (table 2). This made it impossible to create a solution by splitting items by diagnosis. Due to the large amount of DIF shown in the SCI items, and the lack of common items this diagnosis was then omitted from the pooled data leaving TBI and stroke for further analysis.

**Table 1:** Number of working categories, misfitting items and location order of items in each diagnosis and in pooled data.

FIM™ **items**	**Pooled data**			**SCI**			**STROKE**			**TBI**			**Pooled data STR + TBI**		
	**Number of categories**	**Misfit**	**Loc**	**Number of categories**	**Misfit**	**Loc**	**Number of categories**	**Misfit**	**Loc**	**Number of categories**	**Misfit**	**Loc**	**Number of categories**	**Misfit**	**Loc**
**Eating**	3	X	1	3		1	3		3	3		2	3		2
**Grooming**	3		2	2		3	3		2	3		10	3		5
**Bathing**	3		11	3		5	3		12	3		12	3		12
**Dressing upper body**	3		5	3		4	3		7	3		9	3		8
**Dressing lower body**	3		12	3		11	3		11	3		11	3		11
**Toileting**	3		10	3		12	3		8	3		8	3		9
**Bladder**	2		8	3	X	7	2		5	3		6	2		7
**Bowel**	3	X	4	3	X	9	2		1	3		1	3		1
**Transfer bed**	3		6	3		6	3		4	3		4	3		3
**Transfer Toilet**	3		7	3		8	3		6	3		3	3		4
**Transfer bath**	3		9	3		10	3		10	3		7	3		10
**Walk/Wheelchair**	3		3	3		2	3		9	3		5	3		6
**Stairs**	2		13	2		13	3		13	2		13	2		13

Loc = Location order Misfit = Misfitting items SCI = Spinal cord injury TBI = Traumatic Brain Injury STR=Stroke  Pooled data=SCI+TBI+stroke

**Table 2:** Items showing significant DIF in pooled data and between stroke and TBI

	**Pooled data**	**Stroke and TBI pooled**
**Eating**	SCI	
**Grooming**	All	X
**Bathing**	SCI	
**Dressing upper body**	SCI	X
**Dressing lower body**	SCI	
**Toileting**	SCI	
**Bladder**	SCI	
**Bowel**	SCI	X
**Transfer bed**	SCI	
**Transfer toilet**	SCI	X
**Transfer bath**	TBI	X
**Walk/Wheelchair**	All	X
**Stairs**	TBI	

Pooled data = Stroke + TBI + SCI

TBI = Traumatic Brain Injury

SCI = Spinal Cord Injury

All = All three diagnoses

X = DIF present

After omitting the data from patients with SCI, thresholds were again examined and collapsed where necessary. All items were collapsed into three categories, except Bladder Management and Stairs, which were dichotomised (see table I). No individual items showed misfit to model but a significant item-trait interaction remained, indicating that the item hierarchy does not remain exactly the same at different levels of the underlying trait. The person separation index was 0.96.

DIF was still found for 6 items (Grooming, Dressing upper body, Bowel management, Transfer tub, Walk/Wheelchair and Stairs). These were split, forming unique items for stroke and TBI and giving a new scale of 19 items. This new scale was then refitted to the Rasch model. The items showed good fit at the individual level, although again the overall item trait interaction showed significant deviation from model expectations (χ^2 ^= 119.160, df = 57, p = 0.000003).

Person separation index 0.96.

This lack of fit indicates some multidimensionality in the data, and thus the formal test of local independence assumption (for a unidimensional scale) was not performed.

### Summed score comparison

**Figure 1:**
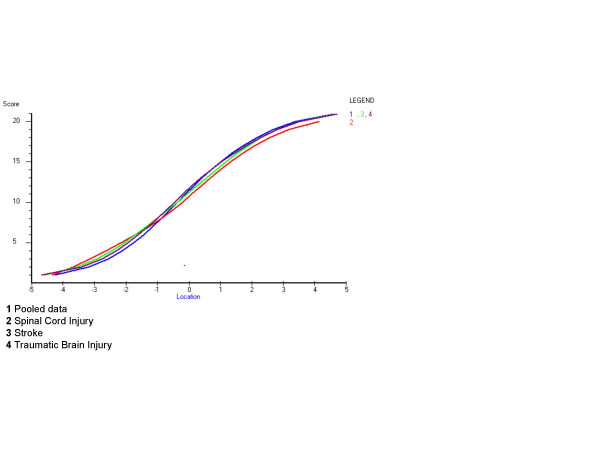
Summed scores after rescoring and their corresponding logit value in the three diagnoses and pooled data.

For the analysis of the clinical meaning of the present DIF an examination of the logit value of the summed score was compared between the diagnoses (not splitting the items). All diagnoses were rescored in the same way for usefulness in the clinical setting, giving three categories in most items, although Grooming, Dressing lower body, Toileting, Bowel and Stairs needed to be dichotomised. This analysis showed small visual differences between the diagnoses as seen in Figure 1. An examination of the differences in logits (table 3) showed no clinical relevance according to the boundaries by Lai and Eton [29].

**Table 3:** Logit values for summed scores after rescoring disordered thresholds

**Sumscore**	**Pooled data**	**SCI**	**Stroke**	**TBI**
**0**	-4,83	-5	-5,35	-4,64
**1**	-3,67	-4,07	-4,14	-3,73
**2**	-2,88	-3,35	-3,31	-3,06
**3**	-2,35	-2,8	-2,73	-2,58
**4**	-1,95	-2,32	-2,27	-2,19
**5**	-1,61	-1,89	-1,88	-1,85
**6**	-1,31	-1,5	-1,53	-1,54
**7**	-1,04	-1,13	-1,2	-1,26
**8**	-0,78	-0,79	-0,89	-0,99
**9**	-0,54	-0,48	-0,59	-0,72
**10**	-0,3	-0,19	-0,31	-0,46
**11**	-0,07	0,08	-0,03	-0,19
**12**	0,17	0,35	0,25	0,09
**13**	0,42	0,61	0,53	0,38
**14**	0,68	0,89	0,81	0,69
**15**	0,96	1,17	1,11	1,03
**16**	1,27	1,48	1,44	1,39
**17**	1,62	1,83	1,81	1,8
**18**	2,04	2,23	2,23	2,26
**19**	2,55	2,75	2,76	2,81
**20**	3,24	3,5	3,47	3,55
**21**	4,16	4,58	4,44	4,54

Pooled data = Stroke + TBI + SCI

TBI = Traumatic Brain Injury

SCI = Spinal Cord Injury

The RUMM 2020 program automatically assigns scores that begin with 0, giving in this case e.g. categories 0, 1, 2 that in RUMM are equivalent to 1, 2, 3 in FIM™ categories. RUMM sum scores ranged from 0–21, which is equivalent to 13–32 in FIM™ sum scores.

## Discussion

In the present study it appears that the 7 category instrument FIM™ t poses several measurement problems. It is shown that a reduction of response categories within each item might be appropriate. A majority of the motor items of the Functional Independence Measure were shown to have cross-diagnostic DIF, meaning that, for example, the Eating item for patients with SCI does not have the same meaning as for stroke or TBI patients. This can influence comparisons between patients in rehabilitation settings. However, appropriate comparison of summed scores with correctly ordered categories seems to be possible as they seem to reflect the same amount of the trait (independence) under investigation. The possibility of sum score comparison could be explained by easier items for some diagnoses possibly being harder for others and vice versa, resulting in the items "balancing out" and the summed level of dependence being the same. This is also one of the purposes of generic instruments: by means of a sum, which should be comparable, to reflect the trait under investigation. Since rehabilitation clinics often have patients with various conditions, it is important that the measures used can be shown to be robust in this way.

In the present study SCI items could not form a construct together with stroke and TBI since there was no linkage item for a Rasch analysis with items split into diagnosis specific items. Questions have been raised about the relevance of the FIM™ in SCI rehabilitation [31] and SCI has previously been treated as a specific group by Wright and co-workers [32]. A new instrument called the Spinal Cord Independence Measure (SCIM) using the FIM™ as a platform has been developed [33] where the authors state that they have refined the items in FIM™ to be more suitable for patients with SCI. Dallmeijer et al. [9] demonstrated DIF in a recent study for seven of the eleven motor items in FIM (Bladder and Bowel management excluded) between patients with stroke, TBI and multiple sclerosis. They used Rasch analysis with the Rating Scale Model (RSM) and anchoring using the threshold measures of the whole group.

The original FIM™ motor scale is not a true ordered category scale and this means there are difficulties in comparing raw sums. The comparison of summed scores done in this study would not have been valid without collapsing the categories. In order to create a scale that was as homogenous as possible using the Partial Credit Model (PCM) a three-category scale was used in the present study for almost all items. However, a few items needed to be dichotomised. Collapsing in this study improved the fit for the diagnoses separately (not shown) and this could imply that a proper order and number of categories might be one way to improve the psychometric property of FIM™.

There may be several reasons for the disordering of categories. Examples are not enough information in the manual, poor definition of categories or training procedures. Different solutions for handling this problem have been suggested. Dallmeijer and co-workers [9] suggested a three-category scale using the RSM. Previous studies of FIM™ from the Pro-ESOR project have suggested a reduction of the scale into four categories for all items using the Rating scale model [34] and as few as two categories for some items using the Partial Credit Model [4,8,22]. Grimby and co-workers used the RSM [35] and suggested a five-category scale. Claesson and Svensson [36] used the rank-invariant statistical method and suggested a scale reduced to four categories, as did also Heinemann and co-workers using Rasch analysis RSM [37]. Thus, a reduction of categories in FIM™ seems to be appropriate, especially taking a modern psychometric approach.

In this study, Eating (pooled data), Bladder (SCI) and Bowel (pooled data and SCI) management did not fit the model despite the collapsing of the categories. Bladder and Bowel management have shown misfit in several studies (e.g. [38] and were referred to by Kucukdeveci and co-workers as an inherent problem [39]. Dallmeijer and co-workers analysed their data without Bladder and Bowel management but also found misfit for Eating in their study [9]. Thus there seems to be an inherent problem with the dimensionality of the scale and this raises fundamental issues about the validity of the 13-item summed score. In the current analysis the item-trait misfit indicated multidimensionality and thus prevented us from doing more formal tests of the local independence assumption.

An idea solution to the presence of DIF by diagnosis (and country) is to allow for the variations that exists across items by splitting items that show relevant DIF and creating an item bank for basic activities of daily living. In an item bank, different subgroups – in this case diagnosis – can have different items but still be compared on the latent trait under investigation, given that there are some common items (unbiased for DIF) to effect the linkage [40,41].

In conclusion, this analysis of the cross-diagnostic validity of the FIM™ shows that care must be taken when data from different diagnoses are pooled. DIF is clearly a problem, but it may be possible to compare group data in a generic instrument such as the FIM™. The continuing misfit of some items in different diagnoses is a concern, as this compromises the validity of the summed score. Thus, when planning interventions (group or individual); when evaluating rehabilitation programs, or comparing patient achievements in individual items, cross-diagnostic DIF must be taken into account.
